# Changes in Arsenic and Copper Bioavailability and Oxytetracycline Degradation during the Composting Process

**DOI:** 10.3390/molecules24234240

**Published:** 2019-11-21

**Authors:** Ebrahim Shehata, Yuanwang Liu, Yao Feng, Dengmiao Cheng, Zhaojun Li

**Affiliations:** 1 Key Laboratory of Plant Nutrition and Fertilizer, Ministry of Agriculture, China-New Zealand Joint Laboratory for Soil Molecular Ecology, Institute of Agricultural Resources and Regional Planning, Chinese Academy of Agricultural Sciences, Beijing 100081, China; ebrahim.shehata@agr.dmu.edu.eg (E.S.); liuyuanwang199121@foxmail.com (Y.L.); fengyao73@126.com (Y.F.); 2 Department of Natural Resources and Agricultural Engineering, College of Agriculture, Damanhour University, Damanhour 22511, Egypt; 3 Research Center for Eco-Environmental Engineering, Dongguan University of Technology, Dongguan 523808, China; chengdm@dgut.edu.cn

**Keywords:** composting, OTC degradation, heavy metal(loid)s, bioavailability, germination index

## Abstract

This research focuses on the effects of the composting process on oxytetracycline antibiotic degradation and the bioavailability of arsenic and copper. A compost experiment was conducted using cow and pig manure contaminated with oxytetracycline, and copper and arsenic salts. The changes in physicochemical properties, oxytetracycline concentration, and the germination index were measured. Copper and arsenic were estimated by sequential chemical extraction. We also detected the effects of compost properties, oxytetracycline concentration, and heavy metal (loid)s on the germination index through simple regression analysis. The results showed that the composting process positively and significantly affected heavy metal(loid)s bioavailability, oxytetracycline degradation, and the germination index. Oxytetracycline concentration declined in all treatments, and the decline was more evident in cows’ manure. The copper and arsenic bioavailable fraction decreased significantly, while the low bioavailability fraction increased. The germination index increased above 50%, which showed that the compost was free of toxic substances. This result also showed that the compost properties had the most significant impact on the germination index, and their regression had the highest R^2^ values (0.84 and 0.99) in the cow and pig manure treatments, respectively. In conclusion, the composting process provides an economical method for oxytetracycline degradation and heavy metal(loid)s bioavailability reduction.

## 1. Introduction

Heavy metal(loid)s pollution is a universal problem because heavy metal(loid)s are nonbiodegradable and bioaccumulate through food chains [1]. Natural sources and human activities are the primary heavy metal(loid) pollution sources [2,3]. In intensive animal production systems, heavy metal additives are used in animal feed to meet mineral nutrient requirements, which lead to increasing concentrations of heavy metal(loid)s in animal waste [4,5,6,7,8]. A small fraction of these heavy metals is absorbed, and the majority is excreted to the environment through the animal’s urine and feces [9]. Heavy metal(loid)s concentrations are increasing worldwide due to the excessive use of low-quality fertilizers, pesticides, sludge, and other biological wastes [10,11]. Heavy metal(loid) accumulation is a limiting factor for the use of organic manure [12,13,14]. In China, crops contaminated with heavy metal(loid)s are estimated at 12 million tons, causing an economic loss of 20 billion yuan annually. These contaminated crops cause many cancers, heart disease, lung disease, and health conditions [15]. Cu is an essential element that has a vital role in plant, animal, and human nutrition [16]. A common practice in animal production is the addition of copper as a feed additive, and most of these additives are excreted by animals (72–80%). Copper addition increases the copper concentration in animal waste and manure [17,18]. Copper and other heavy metals show antimicrobial activity and are toxic toward embryos of *Gobiocypris rarus* and *zebrafish* [19,20,21]. Excessive Cu can also harm livestock and humans [22]. Arsenic (As) has been used since the mid-1940s to control diseases in pigs and poultry. Although it has been banned in European countries, it is still applied in the United States and China [23]. As is a metalloid present in both mineral and organic forms [24]. Organic As is absorbed poorly, resulting in low bioavailability values [25]. Organic arsenic compounds are mineralized by both photolytic and microbial processes during composting [26]. The use of arsenic antibiotics in poultry production causes arsenic contamination. After adding the contaminated animal waste to the soil, plants absorb these minerals. Arsenic then reaches humans bodies through the food chain [27]. So, heavy metals, like Cu and As, have been a big problem that affects the use of manure as organic fertilizer. The total heavy metal(loid) content does not provide enough information about its environmental risks [13,14], and the bioavailability of heavy metal(loid) is an important index. Therefore, we intended to investigate the bioavailability of Cu and As during composting in an attempt to make the composting products harmless. At the beginning of the 20th century, antibiotics began to be used in animal production [28]. An American Cyanamid publication in 1950 reported that antibiotic addition to animal feed had a beneficial aspect. Since then, the addition of antibiotics to animal feed has become more frequent [29]. Antibiotics production was valued at 4.1 billion dollars in 2018 [30]. Tetracycline antibiotics, such as oxytetracycline (OTC), are dominant and widespread in the production of cattle, pigs, and poultry [31,32]. Large fractions of antibiotics are excreted in their original form due to incomplete absorption in the intestines of animals. Their level of urine and animal feces ranges from 10% to 90% [33,34,35,36,37,38]. Animal manure (as fertilizers) spreads easily in the environment [39,40,41]. The antibiotics concentration in animal manure varies between 1 and 10 mg/kg [42] and it reaches hundreds of mg/kg in China [43,44]. Animal antibiotics are more dangerous to the environment than human antibiotics [45] and may alter the microbial community during composting [46]. Composting is an economical method for reducing antibiotic concentrations in manure before application to the land [47,48,49,50,51]. The composting process converts organic waste to mature manure (compost) with aerobic microorganisms, which is the most acceptable and economical method [52]. The thermophilic phase of the composting process generates metabolic heat, which destroys pathogens and reduces the concentration of hazardous organic pollutants [53]. Compost maturity generally relates to the agricultural value and its effect on the soil and plants [54]. Maturity is a term used to specify the level of the phytotoxic substance in composting samples and it is a measure of the completeness of composting [55,56]. The germination index is a maturity test based on seed germination and initial plant growth using a liquid extract from the compost [57]. It is used to measure the phytotoxicity and verify the state of maturity [58,59]. The germination rate test has many advantages; it is simple, inexpensive, requires few samples, no season, and requires only a relatively small sample [60]. These methods have been identified as suitable on-site parameters to establish the endpoint of the composting process [54]. Zucconi et al. [61] reported that the germination test is an easy test that is highly efficient and reliable. It is also suitable for evaluating all types of compost. Unfinished compost has phytotoxic compounds that inhibit seed germination [62]. Compost is considered mature when the germination index is higher than 60%, compared to a control with distilled water [63].

The composting process decreases heavy metal(loid) bioavailability, i.e., the water-soluble fraction, which is the most biologically active fraction [64,65]. Composting is the safest, easiest, and most environmentally friendly way to manage animal waste [66] and it accelerates antibiotic degradation before adding the compost to the soil [67]. During the composting process, antibiotics show the complexation behavior and are usually degraded by microbes and heat decomposition. The combination of oxytetracycline with metal ions (OTC–metal complex), such as copper, significantly affects antibiotic transport and bioavailability during composting [19]. The formation of the OTC-metal complex becomes vital at higher concentrations [19]. Copper addition during composting increases the degradation process of oxytetracycline due to the catalytic effect of copper [19]. Many environmental studies have researched pollutant behavior in various animal wastes. These studies are concerned with each pollutant individually and its changes during composting and the impact on the environment, plants, and microbial communities. However, these pollutants do not exist singly in nature but are combined and mixed. Therefore, our study and experiment followed the changes in copper, arsenic, and oxytetracycline separately and mixed during composting. The objective of this paper was to investigate oxytetracycline degradation during the composting process of pig and cow manure with or without the presence of Cu and As, and the changes of Cu and As bioavailability during the composting process of pig and cow manure.

## 2. Results

### 2.1. Temperature

**Figure 1:**
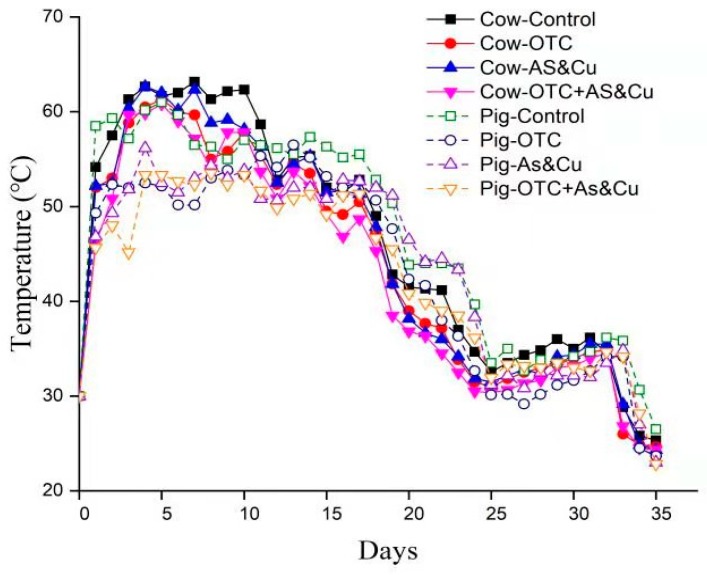
Temperature change in the different treatments during the composting process.

The temperature during the composting process is illustrated in Figure 1. In the experimental units, the temperature increase was evident starting from the first day. The temperature values in cow manure were higher than those in pig manure. We observed a rapid rise in temperature exceeding 50 °C during the first two weeks. After that period, the temperature decreased but was still higher than 30 °C. On the seventh day, the Cow-Control treatment recorded the highest temperature value (63.2 °C) followed by Cow-AS-Cu (62.3 °C). In pig manure, the highest value was 56.5 °C in Pig-Control, and the lowest was 50.2 °C in Pig-OTC. The trials reached maturity on days 33 and 34 for cow and pig manure, respectively. OTC treatments with arsenic and copper recorded the lowest temperature values. Low temperature values were found in the Cow-OTC + AS-Cu and Pig-OTC + As-Cu treatments.

### 2.2. Physiochemical Properties

**Figure 2:**
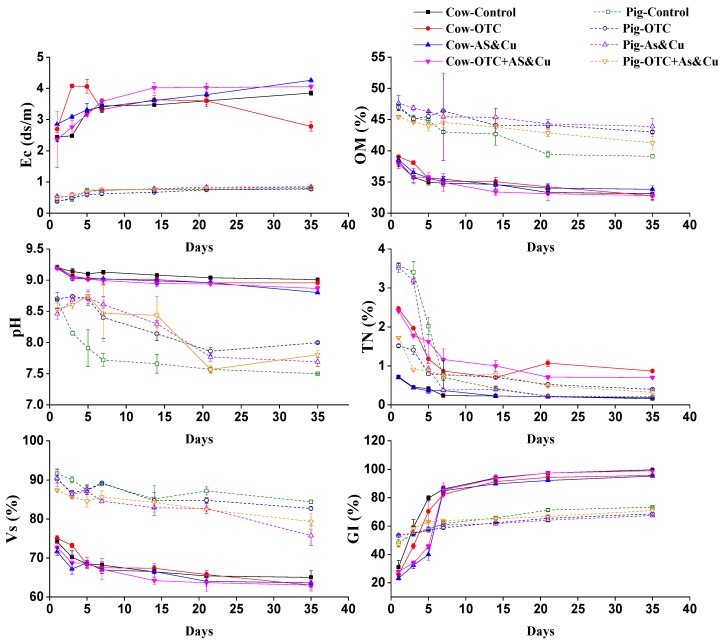
Changes in physicochemical properties and the germination index during the composting process in different treatments.

Figure 2 shows the changes in physicochemical properties during the composting process. The electrical conductivity increased due to organic matter decomposition. The electrical conductivity values increased over time. The treatment with copper and arsenic in cow manure recorded a high value (4.2.6 ds/m) compared to other treatments. Cow manure was more alkaline than pig manure. We also discovered a significant decrease in pH values over time. On the last day, the lowest values of pH were recorded. The lowest pH value in cow manure was 8.8 in Cow-AS-Cu, and in pig manure it was 7.5 in Pig-Control. Volatile substrates (VSs) declined significantly during the experimental period. The highest reduction was in all experimental units during the first week corresponding to the high temperature values. Pig manure values were more significant than cow manure values, illustrating the difference in the compost materials. The highest values for volatile substances were as follows: 91.64 > 90.55% for Pig-Control > Pig-OTC in pig manure and 75.06 > 74.2% for Cow-OTC > Cow-Control in cow manure. The total organic matter decreased significantly during the composting period because of carbon transformation. The organic matter content in the cow manure was lower than that in the pig manure. The microbiological decomposition of the organic matter and the organic nitrogen mineralization was higher in the compost during the initial days than during the final days. Arsenic and copper treatments negatively affected the mineralization process. However, the reduction in cow treatments was less than that in the pig treatments. The TN showed a decreasing trend after the first day.

### 2.3. Changes in the As and Cu Fractions

**Figure 3:**
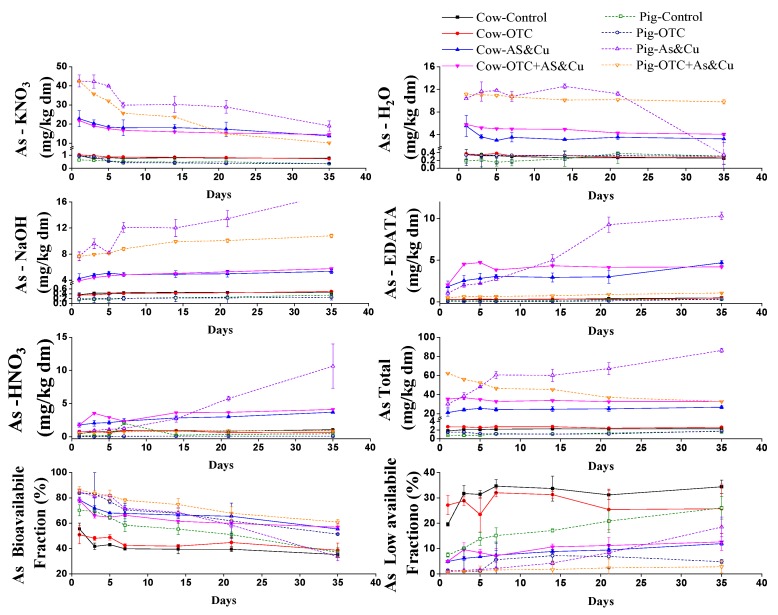
Changes in concentrations of different arsenic extracts during the composting process.

**Figure 4:**
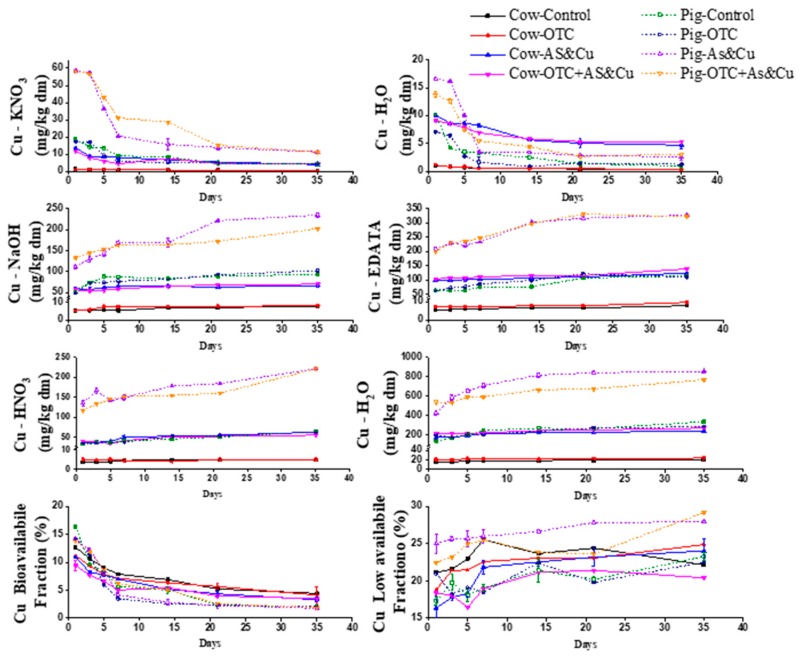
Changes in the concentrations of different copper extracts during the composting process.

Figure 3 and Figure 4 show the changes in the concentrations of the different copper and arsenic extracts over time during cow and pig manure composting. In all experimental units, lower values of the first extract (X-KNO_3_) were found due to the effect of the composting process. In cow and pig manure, the first extract of copper (Cu1 = X-KNO_3_) decreased with time. In pig manure, the arsenic value in the first extract was much higher than that in cow manure. The first extract in the cow and pig manure decreased with time. In the pig and cow manure, the second extract (Cu2 andAs2 = X-H_2_O) significantly decreased over time. The decrease in the Cu extracts (Cu1 and Cu2) was more significant than that of the As extracts (As1 and As2). There was an increase in the ratio of copper and arsenic in the third extract (reducible fraction = X-NaOH = As3 and Cu3) over time. The highest values of the fourth fraction (oxidizable fraction = X-EDATA = As4 and Cu4) were recorded on the final day. The X-NaOH and X-EDATA fractions were available under extreme reduction conditions. Cu was bound firmly in the fourth fraction (organically complexed) and was released slowly over time. The fifth extract (residual fraction), increased during the composting period. This fraction (Cu5 and As5 = sulfides fraction) was never available to the plants. The total concentration of Cu and As in pig and cow manures increased over time until the end of the composting process. The highest copper and arsenic values were recorded after maturity in the fourth treatment (Cow-OTC + AS-Cu and Pig-OTC + AS-Cu).

### 2.4. Bioavailability of As and Cu

Figure 3 and Figure 4 show the changes in the bioavailability of arsenic and copper during the composting process. The bioavailable arsenic fraction decreased with time. In pig manure, the bioavailable fraction of As was significantly higher than that in cow manure. On the first day, the bioavailable fraction of As was more than 70% and then it decreased with time. The Pig-OTC + AS-Cu treatment recorded a 60.8% bioavailable fraction of As, while the other treatments had values less than 38%. Arsenic bioavailability values in Cow-OTC + AS-Cu and Pig-OTC + AS-Cu were higher than those in the other treatments. However, Cow-OTC + AS-Cu and Pig-OTC + AS-Cu treatments were still more toxic than the rest of the treatments because of the increased As availability. The Cu bioavailable fraction decreased during composting. It was lower than the arsenic bioavailable fractions. The Cu bioavailable fractions in cow and pig manure were almost identical. The Cu bioavailable fraction had its lowest values in the Cow-AS-Cu and Pig-AS-Cu treatment. The low-bioavailability fraction of copper increased with time due to the transformation of the copper from the available to the unavailable fraction. In pig compost, the values for the biological availability of copper remained steady in the last days of the treatment. The low bioavailability fraction of arsenic increased with the time because As switched from the available to the non-available form. On the first day, the low bioavailability fraction of arsenic was higher than 50% in the cow manure. After the composting process, it was less than 39% in the case of the Cow-Control and Cow-OTC treatments and was over 55% in the Cow-AS-Cu and Cow-OTC + AS-Cu. The Cow-OTC + AS-Cu treatment recorded lower values than the other treatments. The low bioavailability fractions of arsenic in Cow-AS-Cu and Cow-OTC + AS-Cu were much lower than those in the Cow-Control and Cow-OTC treatment. In pig manure, the percentage of low bioavailability As was smaller than that in cow manure, and its values increased over time. The low-bioavailability As values of the Pig-OTC and Pig-OTC + AS-Cu treatments were lower than those in the Pig-Control and Pig-AS-Cu treatments. The amount of the low bioavailability of copper of the cow manure increased with time. The Cow-OTC + AS-Cu treatment was the lowest. In the pig manure treatments, the Pig-Control treatment was the lowest. The Pig-OTC + As-Cu treatment was the highest, suggesting that the combination of OTC and copper increased the value of low bioavailability Cu.

### 2.5. OTC Degradation during Composting

**Figure 5:**
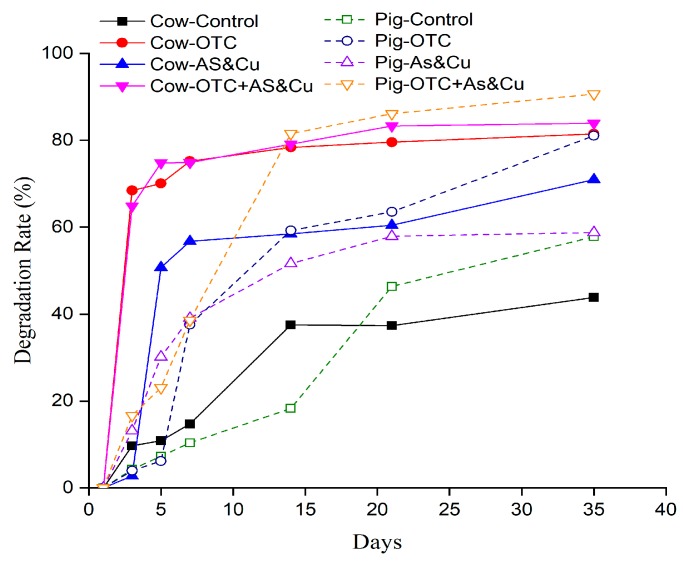
Degradation of oxytetracycline during the composting process.

Figure 5 shows the degradation of OTC throughout the composting process. The results showed that the OTC concentration in pig manure was more than double the concentration in cow manure on the first day. In cow manure, the antibiotic degradation rate on the first day was 9.7%, 68.4%, 2.8%, and 64.9% in the Cow-Control, Cow-OTC, Cow-AS-Cu, and Cow-OTC + AS-Cu treatments, respectively. The rate of the Cow-Control treatment was the lowest. On the third and fifth days, the rate in the Cow-OTC + AS-Cu treatment was the highest (64.9% and 74.8%, respectively). The degradation rate increased with the composting time. On the final day, the control still had the lowest degradation rate (43.9%) and the fourth treatment had the highest (83.9%). The antibiotic degradation rate on the first day in pig manure was lower than that in cow manure. The degradation rate on the final day was 57.8%, 81.1%, 58.8%, and 90.7% in the Pig-Control, Pig-OTC, Pig-AS-Cu, and Pig-OTC + AS-Cu treatments, respectively. On the first day, the degradation rates of the control and OTC treatments were approximately 4% while those of the Pig-AS-Cu and Pig-OTC + AS-Cu treatments were 13.2% and 16.6%, respectively. The fourth treatment on the final day had the highest degradation rate, with a value of 90.7%. The second and third treatment rates had equal degradation rates. The degradation over time in the oxytetracyclines treatment with metal supplements was the fastest. Table 1 shows the different oxytetracycline degradation equations, the degradation constant, the half-life (T_0.5_), and the time to 90% reduction (T_0.9_) throughout the composting process. The degradation constant (Kd^−1^) in pig manure was the highest in the Pig-OTC + AS-Cu treatment. The Pig-Control and Pig-AS-Cu treatments had the same degradation constant value (0.025 d^−1^). The degradation constant of the oxytetracycline treatment (Pig-OTC) was equal to 0.047 d^−1^ while the highest degradation constant value was found in Pig-OTC + AS-Cu (0.068 d^−1^). The constant value increased with the presence of As and Cu. The constant values in the cow treatments were the lowest in the Cow-Control treatment (0.017 d^−1^) and the highest in the Cow-OTC + AS-Cu treatment (0.052 d^−1^). In the control treatment, the half-life was 28.1 to 42.05 days while the lowest half-life (10.2–13.3 days) was recorded for Pig-OTC + AS-Cu and Cow-OTC + AS &Cu, respectively The half-life (T_0.5_) values in the cow manure treatments were 42 days for Cow-Control, 14.4 days for Cow-OTC, 19.6 days for Cow-AS-Cu, and 13.3 days Cow-OTC + AS-Cu while in the pig manure treatments the values were 28.116 days for Pig-Control, 14.6 days for Pig-OTC, 27.4 days for Pig-As-Cu, and 10.2 days for Pig-OTC + As-Cu. The oxytetracycline in the cow and pig manure had the same half-life values. The half-life values in the Cow-OTC + AS-Cu and Pig-OTC + AS-Cu treatments were significantly lower than those in the OTC-only treatment. The time to 90% reduction (T_0.9_) in the control treatments was the highest. The lowest T_0.9_ values were in the Cow-OTC + AS-Cu treatment and the Pig-OTC + AS-Cu treatment. The control treatment value in cow manure was higher than that in pig manure. The lowest T_0.9_ value was found in the Pig-OTC + AS-Cu treatment (34 d).

### 2.6. The Germination Index (GI %)

The germination index increased during composting. Figure 2 shows that the germination rate on the first day was higher in pig manure than that in cow manure. In the cow manure, the fourth treatment had a higher GI than the third treatment. After the seventh day, GI values in all treatments were higher than 80%. On the first day, the GI in all the cow experimental units was less than 50%. the GI values gradually increased over time to register values higher than 95% by the end of the composting process. The GI values in the pig manure treatments were greater than 65% by the end of the composting process. In all pig manure treatments, the germination rate values did not reach 80% by the end of the composting process.

### 2.7. Linear Regression Analysis for the Germination Index

A simple linear regression analysis was performed between GI and various compost characteristics, such as electric conductivity(EC), pH, organic matter (OM), volatile substrates (VS), total nitrogen (TN), and the arsenic and copper extracts. The first equation shown in Table 2 describes how the germination index relates to the compost characteristics. The organic matter had primary significance in the cow manure compost while the pH value had no significant impact. The germination index will be increased 18.2 times if the organic matter increases by 100%. In the pig compost, values of EC, OM, VS, and TN had a significant effect on GI while the pH value had no significant impact. The relationship between the copper extracts (the total concentration and the different extracts) and the germination index is presented in the second equation. In cow manure treatments, all the inputs had a significant effect except the first extract (X-KNO_3_). In the pig manure treatments, the second extract of copper (the water-soluble extract) was the most significant. The third equation describes the relationship between the different As extractions and GI. In the cow manure treatments, the second (X-H_2_O) and fifth extracts (X-HNO_3_) were the most significant. In the pig manure treatments, the first, second, and fourth extracts were the most significant. The third extract and the total concentration did not have any significant effect. The relationship between the germination rate and the OTC concentration was significant and recorded the lowest R^2^ values in pig and cow compost. This relationship is illustrated in the fourth equation.

## 3. Discussion

During the composting process, the temperature increased sharply on the first day. Temperature values in cow manure treatments were higher than those in pig manure treatments. The Cow-OTC + AS-Cu and Pig-OTC + AS-Cu treatments recorded the lowest temperature values. This indicates that mineral additives (As and Cu) and OTC had a negative effect on the temperature increases, which may be caused by the microbial activity. Temperature is considered the main parameter of composting process success [65,68]. The pile temperature stayed above 50 °C for approximate 20 days, showing the harmlessness of the present composting experiment. As for EC cow treatments, copper and arsenic had the highest value (4.2.6 ds/m). Pig manure treatments recorded the lowest EC values. The EC value increased remarkably over time, which agrees with the results of previous studies [69,70,71]. pH is considered the main variable for controlling ion exchange, reduction/oxidation, adsorption, and complexation reactions [54,60]. The cow manure was more alkaline than the pig manure, which may partially explain the different bioavailability of As and Cu in cow manure composting and pig manure composting. We also discovered a significant decrease in pH values over time. The pH values of the Cow-OTC + AS-Cu and Pig-OTC + AS-Cu treatment were higher than those of the control treatments, indicating decomposition and acid formation. This lower value was due to As, Cu, and OTC application. These changes significantly affect heavy metal (loid)s bioavailability [13]. However, Zorpas et al. [72] reported that pH changes during organic matter decomposition due to acid formation. pH is considered as the main variable for controlling ion exchange, reduction/oxidation, adsorption, and complexation reactions [63,68]. Volatiles substrates (VSs) declined significantly during the experimental period, with a reduction percentage of volatiles substrates. Pig manure values were more significant than cow manure. The results agree with those of Hanc et al. [73], who reported that VS content decreased gradually during composting. VS values in the Cow-OTC + AS-Cu and Pig-OTC + AS-Cu treatments were lower than those in control treatments, and lower than those in OTC treatments due to As, Cu, and OTC application. The majority of the volatile solid’s reduction took place during the first weeks [74]. The total organic matter decreased significantly during the composting period because of carbon transformation. This agrees with the findings of Chai et al. [53] and Wei et al. [75]. The organic matter content in the cow manure was lower than that in the pig manure. This highlights the microbiological decomposition and biochemical process, which oxidizes organic C to CO_2_ [70]. The rates of the microbial decomposition of organic matter and the conversion of organic N to its mineral form were higher in the initial days compared with the final days, which was due to the negative effects of arsenic and copper on the mineralization process. It was noted that the reduction in cow manure treatments was less than that in pig manure treatments. The TN showed a descending trend from on the first day. These results are consistent with the results of the research carried out by Hanc et al. [73]. The concentrations of different copper and arsenic extracts changed over time in the cow and pig manure compost. Fuentes et al. [76] reported that the X-H_2_O fractions Pb and Ni were low in composted sludges. Castaldi et al. [77] declared that the water-soluble fraction of Cd, Zn, and Cu gradually decreased in mature compost while Miaomiao et al. [78] showed that the first and second fractions of Cu (Cu1, Cu2) decreased during pig composting. Cu bound strongly to the fourth fraction (EDTA fraction) and released slowly over time during the composting process [79]. Hsu and Lo [13] found that the highest amount of Cu was in the EDTA fraction (Cu4). Nomeda et al. [80] reported that Cu was immobilized during the composting process from mobile fractions to organic and carbonate fractions. Gupta and Sinha [81] revealed that Cu was bound to organic and carbonates fractions. Singh and Kalamdhad [64] reported that the organic and residual fractions (EDTA and HNO_3_ fraction) were more than 50% and 30% of the total Cu, respectively, and they were the most common speciations during composting processes. The total concentration of copper and arsenic in pig and cow compost increased with time. The increase in metal content after composting is illustrated by the mineralization process and the demineralization process [81,82]. The bioavailable fraction is the most biologically available form that significantly affects plants and the environment. It decreased during composting [14]. This effect minimizes the negative impact of metals in the environment. During our experiment, arsenic and copper bioavailability decreased. Arsenic bioavailability values in Cow-OTC + AS-Cu and Pig-OTC + AS-Cu were higher than those in the other treatments, so it was more toxic because of the increased As availability. The lowest fraction was the nonbiological fraction, which represents the parts that are the least available parts to plants [14]. The low bioavailability fraction of arsenic and copper increased with time due to the transformation of copper from its available form to its unavailable form [70,72]. The Cow-OTC + AS-Cu treatment recorded lower values of low availability As than the other treatments. OTC had a positive effect on reducing the low bioavailability As. Values of low availability As increased over time in the pig manure treatment. The values of low bioavailability As in the Pig-OTC and Pig-OTC + AS-Cu treatments were lower than those in the Pig-Control and Pig-AS-Cu treatments. We noticed that the values of low bioavailability As in Pig-OTC + AS-Cu were lower than those in Pig-AS-Cu. This indicates that OTC with As and Cu in pig manure reduced low bioavailability As more than As and Cu treatments individually. The values of the low bioavailability of the copper in the cow manure treatments increased with time. Cow-OTC + AS-Cu values of low-bioavailability copper were lower than those of the rest of the treatments. In the pig manure treatments, the value of low bioavailability copper in the Pig-Control treatment was the lowest. The values of low bioavailability copper in the Pig-AS-Cu and Pig-OTC + AS-Cu treatments were higher. The value of low bioavailability copper in the Pig-OTC + AS-Cu treatment was the highest, suggesting that the combined addition of OTC with copper increased the amount of low bioavailability copper and thus reduced manure toxicity. Miaomiao et al. [78] reported that the bioavailable fraction is the most critical because it is influential and dangerous. The thermophilic phase negatively affects the exchangeable fraction. The bioavailable fraction of heavy metals decreases with composting, as reported by Irshad et al. [65]. Venkateswaran et al. [83] found that the first and second fractions, which are the more bioavailable fractions, are present in large amounts. Xuejiang et al. [84] reported that the bioavailable fractions of Cu mainly transform into low availability fractions during the composting process. The bioavailable fraction of Cu decreased during composting. Cu bound strongly to the fourth fraction and released slowly over the time during the composting process [79]. Chen et al. [85] showed that the composting process affects the availability of heavy metals during the thermophilic phase. Amir et al. [14] reported metal and humus complexation by transformation to the organic fraction (X-EDTA + X-NaOH) during composting. The humification degree has an effect on the speed of heavy metals binding to carboxyl and phenol groups in compost [86]. The OTC concentration declined during the composting process in all treatments. Degradation occurs in the thermophilic phase. The degradation rate was very clear in cow manure compared to that in pig manure. The degradation rate was higher in the early days. The degradation rate in oxytetracycline treatment with metal supplements over time was greater than that in the rest of the treatments. This indicates that the mineral additives of arsenic and copper combined with oxytetracycline had a clear effect on the degradation process. The degradation process during the composting process follows the model of the first kinetic order as described in Equation (1) [66,86,87]

(1) $$C=C_{0}\cdot e^{-K}$$

The fourth treatment had the highest degradation rate on the final day. The high removal of oxytetracycline during the composting process was due to the biological activity and high temperatures, which significantly accelerate OTC degradation [48]. Abiotic and biotic mechanisms were responsible for OTC removal during manure composting with total removal >90% [32,33,35,88,89]. The degradation constant (K) was 0.02 to 0.067 d^−1^ in pig manure and 0.02 to 0.052 d^−1^ in cow manure. That was within the range reported by Alvarenga et al. [90] while Arikan et al. [35] reported that it was 0.012 d^−1^. The degradation constant (K) in the pig manure treatment was highest in the Pig-OTC + AS-Cu treatment. The constant values in the cow treatments were the lowest in the C0 treatment, and the highest was in the Cow-OTC + AS-Cu treatment. The value of the constant increased with the presence of As and Cu. Oxytetracycline treatment in cow and pig manure had the same half-life values of OTC. It is clear that the addition of copper and arsenic with OTC reduced the half-life value and increased the degradation constant value. The lowest values of the time for 90% reduction (T_0.9_) were in the Cow-OTC + AS-Cu and Pig-OTC + AS-Cu treatments. During the composting process, the OTC half-lives varied from 1.56 to 8.50 days. Cu^2+^ as a catalyst accelerated OTC degradation during composting [19]. The T_0.5_ of the OTC treatment during composting was 5.5 days, and the T_0.9_ of the OTC treatment was 18.4 days. However, the T_0.5_ and T_0.9_ values were 29.3 and 97.3 days, respectively, for the control samples [53]. Previous results showed that OTC breaks down more slowly in cow manure than in pig manure, where it persists in cow manure [91]. The germination rate as a maturity index on the first day was higher in pig manure compost than in cow manure compost. The changes that occurred during the thermal phase had a positive effect on increasing GI and decreasing compost toxicity. Pig manure contains more toxic material than cow manure, which affects GI. The biological transformations carried out during the composting process have a significant effect on the toxic substances in manure. Our experimental units recorded GI values of more than 65% and 95% in pig and cow manure, respectively. From the above, it is evident that the composting process had a positive effect on manure toxicity reduction and the manure became mature. A GI value above 50% indicates that the manure became mature due to metabolization of the phytotoxic compounds [92] while Michalopoulos et al. [68] showed that if the value of the germination index was over 70%, the compost was free of phytotoxic substances. In the linear regression analysis, the GI was used to evaluate the toxicity and maturity of the compost [60]. The results showed that the physicochemical properties of the compost, as well as the water extracts of copper and arsenic (the biologically available fraction), had substantial effects on the GI values. The different extracts of copper and arsenic had a more significant effect on GI than their total concentration. The water-extractable Cu (the second extract) in the cow and pig manure treatments was the most influential on the germination index values. This result is consistent with what was reported by Pampuro et al. [93].

The OTC concentration had the least effect on GI due to its breakdown during the early days of the composting process. The results indicated that the first equation for the cow manure compost was the most descriptive. It had the largest R^2^ value (0.84). In the pig manure compost, the first equation R^2^ was 0.99. These values indicate that the compost properties had the most significant effect on the germination index.

## 4. Materials and Methods

### 4.1. Composting Process and Sampling

Fresh cow and pig manure with a low concentration of heavy metals and antibiotics were collected from Zhong Yang country, Lv liang City, Shanxi Province, China. Before starting the experiment, the physiochemical properties and total copper, total arsenic, and OTC were analyzed. The properties of the cow and pig manure, respectively, were: Electrical conductivity (Ec) = 2.44 and 0.74 ds/m; pH = 9.04 and 8.68; organic matter (OM) = 38.58% and 47.65%; total nitrogen (TN) = 0.72% and 3.58%; total copper (CuT) = 19.82 and 140.81 mg/kg d.m; total arsenic (AsT) = 1.80 and 0.75 mg/kg d.m; and oxytetracycline (OTC) = 0.077 and 0.253 mg/kg. The experiment was on cow and pig manure composting with OTC antibiotic (160 mg/kg), and arsenic, and copper salts (Na_3_AsO_4_.12H_2_O = 112.5 mg/kg and CuSO_4_.5H_2_O = 662.5 mg/kg). It included four experimental units for each animal manure. The treatments were: 1. Control (Cow-Control and Pig-Control); 2. Antibiotic treatment (Cow-OTC and Pig-OTC); 3. Heavy metal(loid)s treatment (Cow-AS-Cu and Pig-AS-Cu); and finally, 4. Antibiotic treatment with heavy metalloids (Cow-OTC + AS-Cu and Pig-OTC + AS-Cu). The ingredients were mixed well and the mixture humidity was adjusted to 60%. Distilled water was added and mixed to ensure homogeneity. Forty kilograms of the mixture were placed into 240 L plastic boxes covered with lids. The boxes were incubated for 35 days. Each treatment was replicated three times. The sampling was carried out on days 1, 3, 5, 7, 14, 21, and 35. The piles were turned every day and the temperature was recorded daily. The samples were air- and freeze-dried to determine their physicochemical properties, OTC concentrations, and As and Cu extractions.

### 4.2. Physicochemical Analyses

To find the mean daily temperature, we consistently monitored the pile temperature daily at 9 am and 4 pm. Dry matter and the moisture contents were analyzed after drying at 105 °C for 24 h [94]. Electrical conductivity (EC) and pH were detected in the supernatant using an EC meter and a pH meter [95]. The total organic matter content (OM %) was measured by ignition loss in samples kept at 550 °C for 12 h [96]. The total nitrogen (TN %) was measured by the Kjeldahl method with a KDY-9820 automatic Kjeldahl apparatus (Ruibang Technology Co., Beijing, China) [97]. The total As and Cu were analyzed by digesting samples with aqua regia (3:1 (*v*/*v*) hydrochloric acid: nitric acid) and determining the concentration using the atomic absorption spectrometry (Analysis &Test Center, IEDA, CAAS, Beijing, China) [98].

### 4.3. Heavy Metal(loid) Extraction

The heavy metal(loid) extraction was carried out using 0.5 g of each manure sample in 50 mL centrifuge tubes. A modified version of a sequential extraction procedure was utilized to fractionate the heavy metal(loid)s. The heavy metal(loid)s were fractionated into exchangeable, adsorbed, organically bound, carbonate precipitated, and residual fractions according to Sposito’s procedure [14]. The As and Cu were determined using atomic absorption spectrometry (Analysis &Test Center, IEDA, CAAS, China). Heavy metal(loid)s bioavailability was calculated as reported by Gul et al. [71].

### 4.4. OTC Extraction and Analysis

Compost samples were extracted to determine OTC on days 1, 3, 7, 14, 21, and 35 as described by Ling-Ling et al. [99]. Briefly, 2 g of sample were placed into centrifuge tubes (10 mL) and extracted with 4 mL of extraction buffer (three times) by vortexing for 10 s followed by sonication for 15 min. After each extraction, the extracts were centrifuged at 2500 r min^−1^ for 10 min, and the supernatants were collected, again subjected to centrifugation at 3000 r min^−1^ for 10 min, filtered through cellulose acetate membrane filters, and determined by HPLC. The degradation rate was calculated with the composting time, as well as the calculation of the half-life (T_0.5_) and the constant rate (K) of OTC in the experimental treatments [75].

### 4.5. Germination Index

The germination index (GI) is a maturity test based on seed germination and plant growth using a compost liquid extract [65]. Wheat seeds (*Triticum aestivum*) were used to test the phytotoxicity according to Araújo and Monteiro [100]. We calculated the GI according to Zucconi [57]. The relationship between GI and various compost characteristics, OTC concentration, and heavy metal(loid)s extracts were evaluated for the toxicity and maturity of the compost using simple linear regression analysis at the 0.05 significance level.

### 4.6. Statistical Analysis

Data were subjected to a one-way analysis of variance. The variance and significant differences were compared. All mathematical and statistical calculations were performed using SAS (9.3). The figures were drawn by Origin Pro (2018) and STATA14 performed the simple regression analysis.

## 5. Conclusions

Composting is the most significant economic and environmental process for animal waste management. It reduces copper and arsenic bioavailability and enhances OTC degradation. High temperatures during compost were a major factor. The temperature rose rapidly during the first two weeks of the composting process. That affected the oxytetracycline degradation and the Cu and As bioavailability. The bioavailable fraction of Cu and As significantly decreased during composting. However, the low-bioavailability fractions of As and Cu increased with time. Oxytetracycline degradation rates were higher in OTC treatments with copper and arsenic than those in the treatment with OTC alone. The germination rate increased over time, which is evidence of the effectiveness of the composting process. Simple linear regression analysis showed that the compost properties had the most significant effect on GI. Even though the changes in the physicochemical properties and compost temperatures in the combined treatments with OTC, copper, and arsenic did not reflect the quality of compost compared with those of the control treatments, this combination led to a significant decrease in the bioavailability of copper and arsenic, as well as an increase in the low bioavailability fraction. OTC degradation was also faster in these treatments. The combined treatments also resulted in higher germination values than the individual treatments of OTC, Cu, and As. From the results obtained, further studies are requested to understand the behavior of mixed contaminants, which is very different from that in individual contaminant treatments.

## Figures and Tables

**Table 1:** Different oxytetracycline degradation equations throughout the composting process.

Treatment	Equation	R²	K (d^−1^)	T_0.5_ (d)	T_0.9_ (d)
Cow-Control	$Y=0.09.e^{-0.1X}$	0.91	0.017	42	139.7
Cow-OTC	$Y=46.8e^{-0.2X}$	0.71	0.048	14.4	47.8
Cow-AS-Cu	$Y=0.13e^{-0.2X}$	0.87	0.035	19.6	65.2
Cow-OTC + AS-Cu	$Y=53.1e^{-0.26X}$	0.78	0.052	13.3	44.1
Pig-Control	$Y=0.34e^{-0.14X}$	0.8	0.025	28.1	93.4
Pig-OTC	$Y=447.3e^{-0.28X}$	0.92	0.047	14.6	48.4
Pig-AS-Cu	$Y=0.33e^{-0.16X}$	0.98	0.025	27.4	90.9
Pig-OTC + AS-Cu	$Y=526.3e^{-0.4X}$	0.91	0.068	10.2	34

**Table 2:** The linear regression analysis for the germination index in relation to the heavy metal(loid)s fractions of Cu and As, and other compost parameters during cow and pig manure composting.

Treatment	Equation	R^2^
**Cow Manure**	$1.GI=730.89^{*}+5.34EC-19.04pH+1.884VS-18.21OM^{*}+15.06TN$	0.84
	$2.GI=60.04-2.21Cu1-13.64Cu2^{*}+10.21Cu3^{*}-2.8Cu4+4.12Cu5^{*}-2.04TCu^{*}$	0.77
	$3.GI=3.3^{*}+5.7As1-20.8As2^{*}-57.4As3-12.8As4+81.2As5+4.8TAs$	0.75
	$4.GI=78.9^{*}-0.7OTC$	0.17
**Pig Manure**	$1.GI=17.5^{*}+7.97Ec^{*}-0.9pH-0.25Vs^{*}-2.6OM^{*}+1.04TN$	0.99
	$2.GI=74.6^{*}+0.029Cu1-2.6Cu2^{*}-0.13Cu3+0.04Cu4+0.17Cu5-0.03TCu$	0.67
	$3.GI=59.08^{*}-1.7As1^{*}-7.5As2^{*}0.6As3-12.7As4*+18.8As5-0.24TAs$	0.41
	4.GI=63.01−0.026OTC	0.14

Statistically significant at a probability level of 0.05 * (omitted).
